# Real-World Data on the Effectiveness of Microporous Polysaccharide Hemospheres for Allowing Even Novice Surgeons to Perform Robot-Assisted Radical Prostatectomy Safely

**DOI:** 10.5152/tud.2023.22242

**Published:** 2023-07-01

**Authors:** Takuma Nirei, Tomoyuki Tatenuma, Kentaro Muraoka, Kota Aomori, Yusuke Ito, Hisashi Hasumi, Narihiko Hayashi, Noboru Nakaigawa, Kazuhide Makiyama

**Affiliations:** 1 Department of Urology, Yokohama City University Hospital, Fukuura, Kanazawa, Yokohama, Japan; 2 Department of Urology, Fujisawa Shonandai Hospital, Takakura, Fujisawa, Kanagawa, Japan

**Keywords:** Hemostatics, prostatectomy, robotic surgical procedures

## Abstract

**Objective:**

Radical prostatectomy can be performed more safely and with fewer complications since the advent of robot-assisted surgery. However, increased bleeding is a concern when robot-assisted radical prostatectomy includes lymph node dissection and nerve sparing. In real-world clinical practice, inexperienced surgeons sometimes perform robot-assisted radical prostatectomy. In this study, we investigated the effectiveness of microporous polysaccharide hemospheres as a local hemostatic agent in robot-assisted radical prostatectomy.

**Methods:**

We retrospectively evaluated 301 patients who underwent robot-assisted radical prostatectomy at our institution between December 2017 and November 2020. The patients were divided into 2 groups according to whether their surgery was performed after the introduction of microporous polysaccharide hemospheres as a local hemostatic agent (group A, n = 140) or before it (group B, n = 161: historical control).

**Results:**

Preoperative androgen deprivation therapy was significantly more common in group A than in group B (23 vs. 11, *P = .*009). Furthermore, surgeons were significantly less experienced (*P < .*001) and the operation time was significantly longer (260 minutes vs. 229 minutes; *P < .*001) in group A than in group B. There was no significant difference in any other patient background characteristics or in the surgical outcomes between the groups.

**Conclusion:**

The use of microporous polysaccharide hemospheres allowed even inexperienced surgeons to perform robot-assisted radical prostatectomy without compromising surgical outcomes.

**Main Points**

- Radical prostatectomy can be performed more safely and with fewer complications since the advent of robot-assisted surgery. However, increased bleeding is a concern when robot-assisted radical prostatectomy includes lymph node dissection and nerve sparing.
- We investigated the effectiveness of microporous polysaccharide hemospheres as a local hemostatic agent in robot-assisted radical prostatectomy.
- In real-world clinical practice, inexperienced surgeons sometimes perform robot-assisted radical prostatectomy, but using microporous polysaccharide hemospheres may allow them to perform this surgery without compromising surgical outcomes.

## Introduction

The operative procedure for radical prostatectomy in prostate cancer has changed over time. Radical retropubic prostatectomy (RRP), laparoscopic radical prostatectomy (LRP), and robot-assisted radical prostatectomy (RARP) have all been performed.

The margin positivity and biochemical recurrence rates for RARP are comparable with those for RRP and LRP.^1,2^ Furthermore, RARP is associated with less bleeding, smaller transfusion volumes, and shorter hospital stays than conventional RRP.^3,4^ There are also reports suggesting that postoperative urinary incontinence resolves earlier after RARP than after RRP or LRP.^5,6^ Moreover, erectile function may be better after RARP than after conventional RRP or LRP.^7-9^ Robot-assisted radical prostatectomy has been widely implemented in recent years because surgery has become less invasive over time and perioperative complications have decreased.

Lymph node dissection (LND) is recommended in patients undergoing radical prostatectomy unless the risk of lymph node metastasis is low, but it increases the operation time, blood loss, and risk of complications such as lymphocele.

In the 1980s, Walsh and Donker reported that sparing the neurovascular bundle (NVB) outside the prostate can preserve sexual function.^10^ Although monopolar or bipolar cautery devices are used at some institutions to dissect the NVB, research in a dog model has shown that the thermal energy associated with these devices damages nerve fibers and significantly reduces erectile function.^11^ It has also been reported that dissection of the NVB without use of an energy device during nerve sparing (NS) surgery restores erectile function at an early stage postoperatively.^12,13^ However, hemostasis may be more difficult than when NS is not performed.

Microporous polysaccharide hemospheres (MPH; C. R. Bard, Inc., New Providence, NJ, USA) are plant-based polysaccharides that have been shown to be highly effective in achieving hemostasis.^14^ Microporous polysaccharide hemospheres absorb water, causing immediate aggregation of platelets, red blood cells, and plasma proteins. Furthermore, the gelatinized, water-containing MPH act as a barrier, accelerating the biological coagulation process regardless of the patient’s coagulation status. Microporous polysaccharide hemospheres are used as an adjunct to hemostasis when control of bleeding via ligatures or conventional procedures are ineffective. However, the effectiveness of MPH in RARP has been investigated only in one small study of 30 cases.^15^

Robot-assisted radical prostatectomy was introduced at our institution in 2014, and we now perform more than 100 of these procedures annually. In real-world clinical practice, inexperienced surgeons sometimes perform RARP, and MPH has been used in RARP since June 1, 2019, whenever LND and/or NS is performed because of concerns about increased bleeding. In this study, we investigated the effectiveness of MPH as a local hemostatic strategy in RARP.

## Material and Methods

### Study Population

The study included 301 patients with a pathological diagnosis of prostate cancer who underwent RARP with LND and/or NS at our hospital between December 2017 and November 2020. The patients were divided into 2 groups: group A, in which RARP was performed after June 2019 (n = 140), and group B, a historical control group in which RARP was performed before the introduction of MPH in May 2019 (n = 161). This study was performed in accordance with the ethical standards laid down in the Declaration of Helsinki, and Yokohoma City University Institutional Review Board approved the study (Approval No. B201200056) and agreed that patient data could be retrieved from the hospital database. Patient consent was obtained via the opt-out route using the hospital’s website and noticeboards.

### Surgical Technique

Most operations were performed via the posterior approach. If intraperitoneal adhesions were expected to be severe, a retroperitoneal approach was used. The decision to perform LND and NS was based mainly on the preoperative clinical stage, prostate-specific antigen (PSA) level, and pathological findings.

Energy devices were normally used to dissect the NVB, but in RARP with NS, dissections were performed using a vascular clip made of hemiacetal. The dorsal vein complex was ligated with running sutures. After removal of the prostate, the pneumoperitoneum pressure was reduced from 10 mmHg to 5 mmHg and hemostasis of the prostatic bed was achieved as an energy device or sutures. The urinary tract was reconstructed by reinforcement of the posterior wall using a Rocco stitch,^16,17^ vesicourethral anastomosis, and reinforcement of the anterior wall with running sutures.

**Figure 1.**
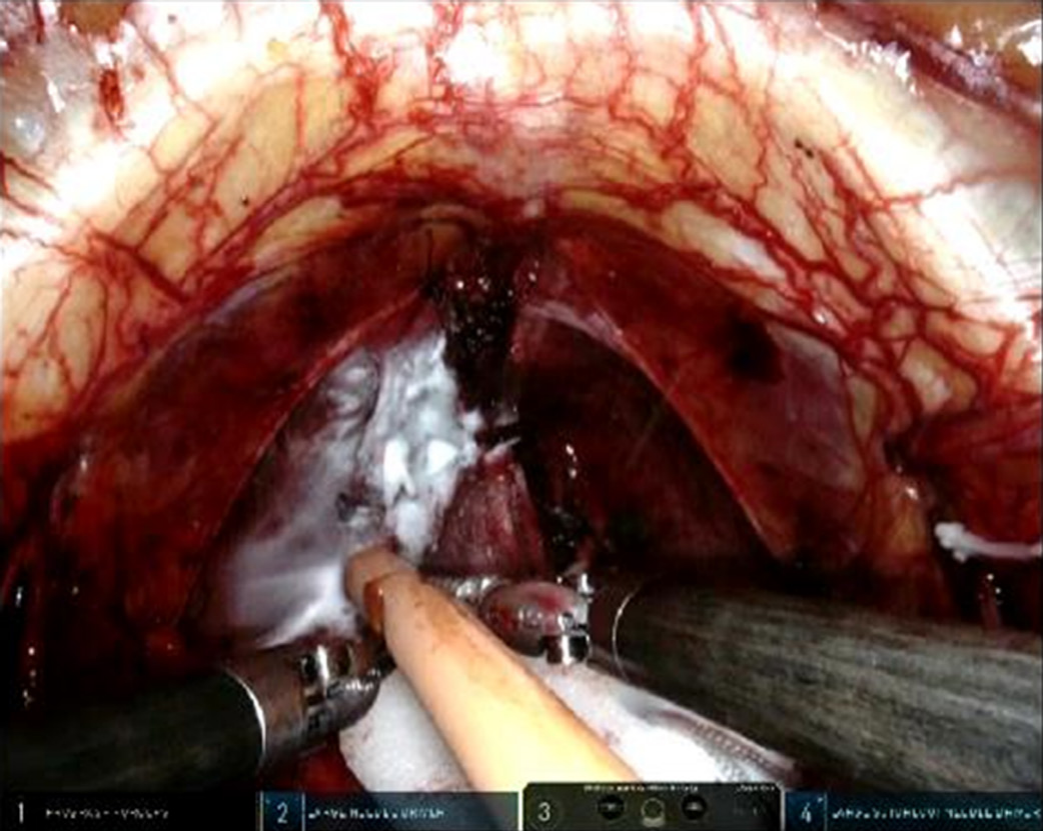
Microporous polysaccharide hemospheres were sprayed on the prostatic bed and on the dissection surface of the prostate and transected edge after lymph node dissection following conventional hemostatic methods in group A.

After conventional hemostatic methods had been performed, a catheter was inserted through the assistant port, MPH was sprayed on the prostatic bed and dissection surface of the prostate, and on the transected edge after LND in group A (Figure 1). Other local hemostatic agents, such as oxidized regenerated cellulose and fibrin sealant, were used at the bleeding site according to the surgeon’s judgment.

### Statistical Analysis

Patients were followed up for 3 months after surgery. Background characteristics (age, height, weight, body mass index, initial PSA [at diagnosis], Gleason score, clinical T stage, clinical N stage, preoperative androgen deprivation therapy [ADT], Charlson Comorbidity Index [CCI],^18^ history of abdominal surgery) and surgical outcomes (LND and NS, weight of specimen, surgeon experience, operation time, blood loss, use of other local hemostatic agents, change in hemoglobin from after surgery to the next morning, total drainage volume, date of drain removal, postoperative complications, intestinal obstruction, and length of hospital stay) were compared between the study groups using the Mann–Whitney *U* test for continuous variables and Pearson’s chi-squared test for categorical variables. All statistical analyses were performed using IBM Statistical Package for the Social Science (SPSS) Statistics for Windows version 20.0 (IBM SPSS Corp., Armonk, NY, USA). A *P*-value <.05 was considered statistically significant.

## Results

Patient background characteristics are shown in Table 1. There was no significant difference in age, height, weight, body mass index, initial PSA, Gleason score, clinical T stage, clinical N stage, CCI, or history of abdominal surgery between the 2 groups. Significantly more patients received preoperative ADT in group A than in group B (23 vs. 11; *P = .*009). A retroperitoneal cavity approach was used in 5 patients (group A, n = 2; group B, n = 3); the between-group difference was not significant (*P = .*768).

Surgical outcomes are shown in Table 2. Lymph node dissection, NS, specimen weight, blood loss, use of other local hemostatic agents, amount of change in hemoglobin, total drainage volume, date of drain removal, complications (including intestinal obstruction), and length of hospital stay were not significantly different between the 2 groups. The number of operations performed by surgeons with experience in less than 40 cases was significantly greater in group A than in group B (71 cases vs. 20 cases; *P < .*001). Also, the operation time was significantly longer in group A than in group B (260 minutes vs. 229 minutes; *P < .*001). No patient in either group required intraoperative transfusion or conversion to open surgery. Postoperative transfusion was required in one patient in group A who underwent dissection of the obturator, internal and external iliac lymph nodes, and the lymph nodes on the anterior surface of the sacrum.

Details of local hemostatic agents used other than MPH are shown in Table 3. There was no significant difference in the frequency of use of oxidized regenerated cellulose and/or fibrin sealant between the 2 groups.

## Discussion

Microporous polysaccharide hemospheres were used in patients who underwent LND and/or NS during our study period because of concerns about increased bleeding. However, the amount of change in hemoglobin and total drainage volume were not significantly different between the 2 groups, possibly because energy devices, such as bipolar clamping or sealing devices, were used during LND to ensure that the margins were reliably treated. Moreover, it is possible that there were few benefits from using MPH when NS was performed and that conventional hemostasis using sutures and vascular clips was sufficient.

Although all operations were performed by the same surgeon^15^ or surgeons with a certain amount of experience (48 cases were excluded from the first operation in order to reduce bias related to the learning curve) in the previous studies,^19^ some surgeons in the present study started performing RARP during the observation period. Three of 6 surgeons in group A and 2 of 5 surgeons in group B treated fewer than 40 cases in total from the first operation. Therefore, surgeons were significantly less experienced (71 cases vs. 20 cases; *P < .*001) and the operation time was significantly longer (260 minutes vs. 229 minutes; *P < .*001) in group A than in group B. Furthermore, significantly more patients in group A received preoperative ADT (23 vs. 11; *P = .*009). The reasons stated above may have contributed to the increases in bleeding and drainage volume. However, there was no significant difference in the frequency of use of other hemostatic agents between the 2 groups, and all operations were performed without compromising surgical outcomes.

While a small proportion of patients in the previous reports required transfusion,^15,19^ none of the patients at our institution required intraoperative transfusion, and only one in group A required postoperative transfusion. It is possible that bleeding would have been less and hemostasis adequate regardless of MPH use.

To our knowledge, this study, which included a total of 301 cases, is the largest to report on the effectiveness of MPH in RARP. Considering that the surgeries were performed by any of the 11 surgeons, half of whom were inexperienced, the study design seems to reflect real-world clinical practice.

This study has some limitations. First, all patient information was obtained retrospectively from medical records and none of the patients were followed up for more than 3 months postoperatively. Second, there were empirical differences between the 2 groups in that there was a difference in the number of cases treated by each surgeon. However, there were more inexperienced surgeons in group A than in group B. Although the patient background characteristics were generally similar between the 2 groups, significantly more patients in group A received preoperative ADT. Moreover, this study was performed at a single institution, and almost all of the patients were Asians. Functional aspects, such as postoperative incontinence and erection status and long-term complications, could not be verified.

In real-world clinical practice, inexperienced surgeons sometimes perform RARP. However, using MPH allowed surgeons to perform RARP without compromising surgical outcomes.

## Figures and Tables

**Table 1.** Patient Background Characteristics

	Group A (n = 140)		Group B (n = 161)		*P*
	Mean/Number	Range/(%)	Mean/Number	Range/(%)	
Age (years)	70	49-79	70	44-82	.198
Height (cm)	168	144-181	167	145-184	.945
Weight (kg)	67	50-98	65	48-101	.068
BMI	23.5	18.8-31.7	24.2	18.4-34.0	.093
Initial PSA	7.60	1.38-114	8.72	3.95-73.2	.068
Gleason score					.065
6	5	(3.6)	8	(5.0)	
7	82	(58.6)	107	(66.5)	
<8	47	(33.6)	45	(28.0)	
Clinical T stage					.491
T1c	12	(8.6)	18	(11.2)	
T2a	69	(49.3)	75	(46.6)	
T2b	13	(9.3)	17	(10.6)	
T2c	32	(22.9)	37	(23.0)	
T3a	9	(6.4)	13	(8.0)	
T3b	3	(2.1)	0	(0)	
Clinical N1	3	(2.1)	3	(1.9)	.863
Preoperative ADT	23	(16.4)	11	(6.8)	.009
CCI (>1)	23	(16.4)	25	(15.5)	.841
History of abdominal surgery	46	(32.9)	45	(28.0)	.355

ADT, androgen deprivation therapy; BMI, body mass index; CCI, Charlson Comorbidity Index; initial PSA, prostate-specific antigen level at the time of diagnosis.

**Table 2.** Surgical Outcomes

	Group A (n = 140)		Group B (n = 161)		*P*
	Mean/Number	Range/(%)	Mean/Number	Range/(%)	
LND					.083
Localized^†^	21	(15.0)	32	(19.9)	
Standard^‡^	64	(45.7)	55	(34.2)	
Expanded^§^	39	(27.9)	61	(37.9)	
NS					.281
Right	11	(7.9)	15	(9.3)	
Left	19	(13.6)	15	(9.3)	
Bilateral	14	(10.0)	9	(5.6)	
Specimen weight (g)	42	20-104	42	16-118	.056
Surgeon experience (<40 cases)^||^	71	(50.7)	20	(12.4)	<.001
Operation time (min)	260	160-397	229	160-356	<.001
Blood loss (mL)	100	<50-1400	100	<50-1500	.350
Other local hemostatic agent used	13	(9.3)	22	(13.7)	.237
ΔHb (g/dL)	0.6	−1.3, 2.7	0.6	−1.8, 2.6	.456
Total drainage volume (mL)	296	4-3130	316	2-4883	.523
Date of drain removal	3	2-7	3	2-7	.142
Complications	10	(7.1)	20	(12.4)	.127
Intestinal obstruction	5	(3.6)	11	(6.8)	.208
Hospital stay (days)	8	7-14	8	7-34	.250

ΔHb, amount of change in hemoglobin; LN, lymph nodes; LND, lymph node dissection; NS, nerve sparing.

^†^Dissection of obturator LN. ^‡^Dissection of obturator and internal iliac LN. ^§^Dissection of obturator, internal iliac, and external iliac LN. One patient in group A underwent dissection of the obturator, internal, and external iliac LN and the LN on the anterior surface of the sacrum. ^||^The number of operations performed by surgeons with experience in less than 40 cases.

**Table 3.** Details of Local Hemostatic Agents Used other than Microporous Polysaccharide Hemospheres

	Group A (n = 140)		Group B (n = 161)		*P*
	n	%	n	%	
Oxidized regenerated cellulose	3	2.1	5	3.1	.604
Fibrin sealant	10	7.1	13	8.1	.762
Both of the above	0	0	4	2.5	.060
